# Livelihood Security and Adherence to Antiretroviral Therapy in Low and Middle Income Settings: A Systematic Review

**DOI:** 10.1371/journal.pone.0018948

**Published:** 2011-05-12

**Authors:** Beth S. Rachlis, Edward J. Mills, Donald C. Cole

**Affiliations:** 1 Division of Epidemiology, Dalla Lana School of Public Health, University of Toronto, Toronto, Canada; 2 Faculty of Health Sciences, University of Ottawa, Ottawa, Canada; University of Cape Town, South Africa

## Abstract

**Introduction:**

We sought to examine the association between livelihood security and adherence to antiretroviral therapy (ARVs) in low- and middle-income countries (LIMC).

**Methods:**

Performing a systematic review, we searched, independently and in duplicate, 7 electronic databases and 2 conference websites for quantitative surveys that examined the association between indicators of livelihood security and adherence to ARVs in LIMC between 2000–2010. Criteria for relevance were applied to complete papers (quantitative study with estimates of associations) and quality assessment was conducted on those deemed relevant. We performed three regressions to measure the association between each type of livelihood and adherence.

**Results:**

Twenty original studies and 6 conference abstracts were included, the majority from Africa (n = 16). Seventeen studies and 3 conference abstracts were cross-sectional and 3 studies and 3 abstracts were prospective clinical cohort studies, with considerable variation in quality for studies of each design type. Among the diverse populations represented, we observed considerable variation in associations between measurements of livelihood indicators and increasingly accepted adherence measures, irrespective of study design or quality. A financial capital indicator, financial constraints/payment for ARV medication, was more commonly associated with non-adherence (3/5 studies). A human capital indicator, educational level, was most commonly associated with adherence (11/20 studies).

**Discussion:**

Additional better quality research examining livelihood security is required to inform provision of optimal supports for adherence and mitigation of the impacts of HIV/AIDS.

## Introduction

The HIV/AIDS epidemic has taken a particular toll on low- and middle-income countries (LIMC), with sub-Saharan Africa heavily affected by both disease and poverty. Among the many challenges faced by clinicians and AIDS organizations are maintaining health in the face of poverty that may preclude access to food and medication adherence [1]. Although several development initiatives have been established by different AIDS organizations, such as micro-finance and support groups, little is understood about the impact of livelihood security and its eventual impact on long-term patient status, including mortality.

Livelihood is closely linked to socio-economic status (SES), a term often used to reflect an individual's access to resources such as food, potable water, health facilities, educational opportunities, and housing [1], [2]. Assets include the types of capital that can be used directly or indirectly to generate livelihoods and reflect natural (e.g., land, water), physical (e.g., infrastructure, roads), financial (e.g., money, savings, income), human (e.g., knowledge, education, ability to work), and social (e.g., networks, kin, membership in a group) forms [3]. A livelihood approach, as a framework, explores how individuals, households, or communities behave under specific conditions, analyzing their ability to cope and adapt in response to external shocks such as drought or civil strife [4], [5].

In the context of HIV/AIDS, there has been growing recognition that the various aspects of livelihoods that increase risk of illness and death need to be identified [1]. Limited livelihood security can lead to engaging in risky behaviours that increase HIV incidence [5]. Among those receiving ARVs, limited livelihoods can reduce adherence, create adverse gastrointestinal and other adverse events due to poor diets, and lead to disrupted medication supplies [1], [5]–[7].

Highly active antiretroviral therapy (HAART) provides the hope that people living with HIV/AIDS (PLWHA) can now live longer [8], [9] and more productive lives. Nevertheless, as of 2008, only 42% of clinically eligible individuals in LIMC were receiving treatment [10], despite the fact that treatment has been recognized as an essential tool for mitigating the impacts of HIV on affected communities [11]. Treatment efficacy with ARVs relies on sustained adherence, critical for viral suppression and the prevention of resistance, disease progression, and death [12], [13]. Unfortunately, adherence remains a challenge for many [14]–[16], given obstacles such as dosing schedules, dietary requirements, and adverse effects [15], [17].

Since the rapid scale-up of ARVs in resource-limited settings, numerous studies have focused on treatment adherence [14], [15], [18]. In 2006, we previously reviewed facilitators and barriers to adherence in developed and developing nations, some of which were livelihood-related (e.g., cost, available social support). However, our review was limited in its ability to directly measure the associations between identified factors and adherence levels [15]. There remain important gaps in our understanding of the relationship between livelihood security and adherence to ARVs, specifically in the context of treatment sustenance. The objective of our review is to evaluate the adherence literature specifically focused on the role of livelihood security on adherence to ARVs in LMIC.

## Methods

### Inclusion Criteria

We aimed to include all observational studies that examined the association between financial, human, and social capital, as important indicators of livelihood security, and adherence to ARVs in LMIC settings.

### Ethics

Ethical approval was not sought for this systematic review as only published data was included. Furthermore, no personal identifiers from patients described in included studies were included. Therefore, written consent from such patients was neither sought nor needed.

### Search Strategy

We searched the following databases: AMED (inception to January 2010), Campbell Collaboration (inception to January 2010), CinAhl (inception to January 2010), CAB Abstracts (inception to January 2010), Cochrane Library (inception to January 2010), Embase (inception to January 2010), and PubMed via Medline (inception to January 2010). Conference abstracts from the International AIDS Society conferences (inception to 2009) and Conferences on Retroviruses and Opportunistic Infections (inception to 2009) were also sought.

Our search strategies combined terms that represented livelihood security and HIV. An initial scan of the literature noted that the majority of potentially relevant studies focused on financial, human, and/or social forms of capital. While the role of natural and physical capital was referred to in the qualitative literature, their association with adherence were infrequently estimated. Therefore, in the present study, we focused solely on financial, human, and social types of capital. Using the UK Department of International Development (DFID) Sustainable Livelihood Framework as a guideline [19], financial capital in the present study denotes access to financial resources; human capital encompasses skills, knowledge, the ability to work, and nutritional factors; and social capital refers to formal and informal social relationships.

As we were interested in the interaction between adherence and antiretroviral therapy, HIV and livelihoods, our search strategy combined terms representing “HIV OR AIDS” AND “adherence to antiretroviral therapy” AND “financial capital OR human capital OR social capital”. We supplemented this search by reviewing the bibliographies of key papers. As the PRISMA Guidelines for Meta-Analyses and MOOSE guidelines for Systematic Reviews of Observational Studies [20] suggest that observational studies are often not indexed well, we did not limit our search by study design.

### Study selection

BR and DCC independently reviewed the abstracts. Initially, eligible studies met the following criteria: (1) reported an original research study; (2) measured adherence to antiretroviral therapy; (3) contained content addressing the association between social, human, or financial capital and adherence to antiretroviral therapy; and (4) was set in a low-income or middle-income country as defined by the World Bank Country Classification [21]. The relevant qualitative studies, though useful with discussions on the contribution of livelihood factors, could not contribute information on the estimate of the association between the livelihood measures of interest and adherence, and, as a result, were excluded.

### Quality Assessment

We extracted data on the quality of included studies using criteria consolidated from existing critical appraisal sources [14], [22], [23]. As many studies were clinical case series, with populations of patients being asked additional questions during regular visits, some criteria relevant for more traditional population surveys were not helpful for our assessment (e.g., representativeness of population, use of random selection). For longitudinal studies we added criteria with respect to follow-up: 1) the proportion followed at each stage of the study was described-(e.g., numbers potentially eligible, examined for eligibility, confirmed eligible, included in the study, completing follow-up, and analyzed) and 2) participants reasons for non-participation at each follow-up were presented [23]–[25]. Conference abstracts usually did not contain sufficient information upon which to conduct quality assessments. Given the limited number of available studies and our interest in exploring the association of livelihood and adherence in a range of LMIC, we chose not to exclude any study based on quality.

### Data Abstraction

When the full-text of an abstract was not available or when information was not available in the full-text paper, we contacted the study authors for additional information. BR initially appraised quality and content and abstracted relevant data. DCC acted as a secondary reviewer. When disagreement occurred we reached consensus through discussion. The reviewers discussed the studies including characterization of different livelihood measures reported, the strengths of different adherence measures, and the patterns of findings encountered. In addition to descriptive material, we abstracted data on prevalence e.g. of other livelihood factors associated with adherence, and the types and magnitudes of associations e.g. of education with adherence, reported in each study.

### Quantitative Data Synthesis

Studies were organized and sorted by year of publication, by study design, by sample size, by response rate, and by the livelihood measures examined. The prevalence of various livelihood measures were determined and the proportion of participants reporting each livelihood factor was also captured from individual studies. Patterns across studies were then examined with respect to the estimates of the given associations as well as the precision around these estimates. While few studies consistently measured the same independent (i.e. livelihood) factors and dependent variable (i.e. adherence) we chose to run three separate meta-regressions for each type of livelihood measure (financial, human, social) as a predictor to determine if there were individual effects on adherence levels. Analyses were performed in STATA. A p-value of less than 0.05 was considered statistically significant.

## Results

### Study Selection and Characteristics

**Figure 1:**
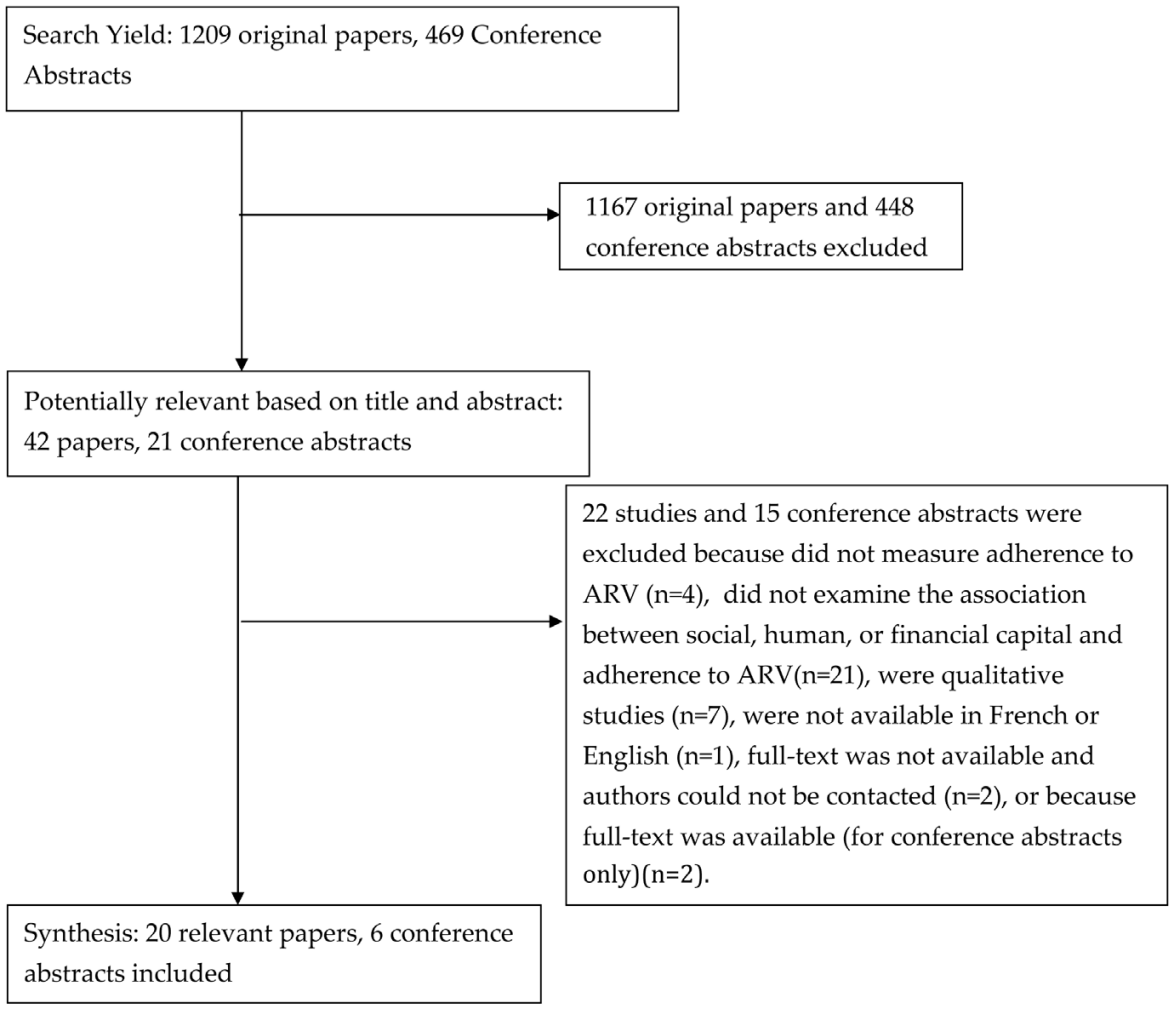
Flow Chart of Studies Included in Review.

The initial literature search produced 1209 papers and 469 conference titles and abstracts. There was near perfect agreement between BR and DCC on choosing the potentially relevant 42 papers and 21 abstracts from this larger set. Of these, 20 papers [26]–[45] and 6 conference abstracts [46]–[51] were judged relevant for our review (See Figure 1). There was perfect agreement on the final papers and abstracts selected (kappa = 1). All were published in English. The majority of included papers initially were identified in PubMed via Medline (n = 15, 75%) [26]–[30], [32], [33], [35], [37]–[42], [45]. Of the remaining 5 papers, 4 were from Embase [31], [34], [36], [43] and one was identified from the CAB Abstracts database [44]. All included abstracts were identified through the International AIDS Society conference abstract database.

All included papers and abstracts employed a quantitative methodology and used structured questionnaires (n = 8) [26], [35], [37], [42], [45], [48], [50], [51] or structured interviews (n = 18) [27]–[34], [36], [38]–[41], [43], [44], [46], [47]. Seventeen of the papers [26]–[42] and 3 of the conference abstracts [46]–[48] were cross-sectional studies and 3 papers [43]–[45] and 3 abstracts [49]–[51] were longitudinal studies, following up patients over time. Almost all studies (n = 19) used logistic regression analysis to measure the association between livelihoods and adherence. One study [43] used Cox's proportional hazards to assess the relative hazard of non-adherence. Although detailed information on the nature of statistical analysis was poorly described, all abstracts reported conducting multivariable analysis.

### Quality Assessment

Tables 1 and 2 display the quality criteria results. There was no improvement in quality over time and no studies reported contacting non-responders. The proportion of studies meeting our quality criteria ranged from 0–100%.

**Table 1:** Quality Criteria for included cross-sectional studies [n = 17, (26–42)].

	Describe setting, time period	Include eligibility criteria, sources, selection	Include sample size calculations	Include and describe response rates	Describe variables outcomes, exposures, covariates	Describe data sources, measures	Survey pre-tested	Survey tool tested for validity	Describe consent process, ethics approval	Train interviewer	Translate survey tool	Describe analysis	Include participant descriptions	Include events or outcome measures	Main results: include un-adjusted and adjusted results	Main results- describe adjustment
Aboubacrine/2007	√	√		√	√	√							√	√		
Boyer/2009	√	√			√	√			√	√		√	√	√	√	√
Byakika-Tusiime/2005	√	√			√	√		√	√	√	√	√	√	√	√	√
Carlucci/2008	√	√	√	√	√	√		√	√		√	√	√			
Iliyasu/2005	√	√	√	√	√	√	√	√	√	√		√	√	√		
Malangu/2008	√	√		√	√	√	√		√	√			√			
Nachega/2004	√	√	√	√	√	√		√	√	√	√	√	√	√		
Nemes/2007	√	√		√	√	√			√			√	√	√	√	√
Pinheiro/2002	√	√		√	√	√		√	√	√		√	√	√	√	√
Ramadhani/2007	√	√			√	√	√	√	√		√	√	√	√	√	
Sarna/2008	√	√	√	√	√	√		√	√	√	√	√	√	√	√	√
Silva/2009	√		√		√	√		√				√	√		√	√
Stout/2004	√	√		√	√	√	√	√	√		√	√	√	√	√	√
Uzochukwu/2009	√	√	√	√	√	√			√	√	√	√	√	√		
Wang/2007	√	√		√	√	√			√			√	√	√	√	√
Weiser/2003	√	√	√	√	√	√	√	√	√	√	√	√	√	√		
Williams/2007	√	√	√	√	√	√							√	√		
TOTAL/17	17	16	8	13	17	17	5	10	15	9	8	13	16	14	9	8

**Table 2:** Quality Criteria for included longitudinal studies [n = 3, (43–45)].

	Describe setting, time periods	Describe eligibility criteria, sources, methods of selection, methods of follow-up	Include sample size calculations	Include and describe response rate	Describe variables-outcomes, exposures, covariates	Describe data sources, survey tool	Survey pre-tested	Survey tested for validity	Describe consent process, ethics approval	Train interviewer	Translate survey tool	Describe analysis	Describe proportion followed at each stage	Include reasons for drop out at each follow-up	Include participant description	Include events or outcome measure	Main results-Include un-adjusted and adjusted results	Main results-describe adjusted
Bonolo/2005	√	√		√	√	√	√		√			√			√	√	√	√
Erah/2008	√	√	√	√	√	√	√	√	√						√	√		
Orrell/2003	√	√			√	√			√			√	√	√	√	√	√	√
Total/3	3	3	1	2	3	3	2	1	3	0	0	2	1	1	3	3	2	2

All cross-sectional studies adequately described the setting, variables included, and data measurement sources, and provided descriptive characteristics of included participants. Ten studies (58.9%) noted use of a previously validated survey [28]–[32], [34]–[38], [41], but few studies reported pre-testing their survey instrument (n = 5, 29.4%) [30], [31], [35], [38], [41]. Nine (52.9%) studies reported both unadjusted and adjusted estimates [27], [28], [33]–[38], [40] and eight (47.1%) justified the inclusion of any covariates [27], [28], [33], [34], [36]–[38], [40] (no studies reported stratification so we only assessed the use of adjustment).

All longitudinal studies described the study setting, the populations sampled, the variables and data measurement sources used, provided information on informed consent, provided descriptive characteristics of participants, and provided data on outcome events and summary measures. One study used a previously validated questionnaire [44] yet none of the two studies conducting interviews provided details on whether the interviewer had been trained. Only one study provided detail on the follow-up of participants and the reasons for losses-to-follow-up [45]. Two studies (66.7%) reported unadjusted and adjusted estimates and also justified their inclusion of covariates [43], [45].

### Settings and Populations (Tables 3 and 4)

**Table 3:** Characteristics of included cross-sectional papers [n = 17, (26–42)] and conference abstracts [n = 3, (46–48)].

Author	Yr	Country	N	Setting	Female (%)	Median Age, y	Response Rate	Assessor	Adherence, % threshold for measurement	Adherence Proportion (%)
**Aboubacrine**	2007	Mali, Burkina Faso	270	Public hospital, community based clinics	65	36–40 (median range)	100	patient	less than 100%, no. doses missed in past 7 days	58.5
**Boyer**	2009	Cameroon	532	Public hospital	70.9	mean (SD): 38 (9)	83.9	patient	high, moderate, low score in past 4 days	56.6
**Byakika-Tusiime**	2005	Uganda	304	ARV delivery centres	53	39	unknown	patient	≥95%, No. doses taken/no.prescribed in last 3 days	68
**Carlucci**	2008	Zambia	409	Rural mission hospital	63	39 (32–47)	78.2	patient	≥95%, No. doses taken/no. doses prescribed for total time	83.7
**Iliyasu**	2005	Nigeria	263	Teaching hospital	34	36.2 (3.3)	94	patient	≥95%, based on previous 7 days	54
**Malangu**	2008	South Africa	180	Hospital	68.8	36.7 (8.1)	63.2	patient	≥95%, No. doses taken/no. doses in past 7 days	57.2
**Nachega**	2004	South Africa	66	public outpatient clinic	77	36.1 (10.1)	Unknown	patient	≥95%, No. doses taken/no. doses in past 30 days	88
**Nemes**	2004	Brazil	1972	Health service sites	38	39.3	97	patient	≥95%, No. doses taken/no. doses in past 3 days	75.1
**Pinheiro**	2002	Brazil	195	Publicly funded specialist clinic	39	35 (17–67)	84	patient	≥95%, No. doses taken/no. doses prescribed in past 2 days	56.9
**Ramadhani**	2007	Tanzania	150	Infectious disease clinic	63	41 (19–69)	Unknown	patient	100, >2 days without dose	84
**Sarna**	2008	India	310	Public and private outpatient clinic	16	36 (23–70)	96	patient	≥90, no. Doses taken/no. prescribed in past 4 days	84
**Silva**	2009	Brazil	412	Clinics at referral hospital	21.8	36 (17–67)	Unknown	patient	≥90, no. Doses taken/no. prescribed in past 5 days	74.3
**Stout**	2004	Costa Rica	88	Social security hospital	15	38.2 (18–79)	87	patient	100, no. Doses taken/no. prescribed past 3 days	85
**Uzochukwu**	2009	Nigeria	174	Teaching hospital	37.5	34.6 (7.2)	95.6	patient	100, miss at least 1 dose in past 30 days	25
**Wang**	2007	China	181	Clinic	59.7	47.8 (11.3)	100	patient	≥95, no. Doses taken/no. Doses prescribed past 3 days	81.8
**Weiser**	2003	Botswana	109	Private clinic	50	Not available	97.3	patient or clinician	≥95, previous year of missing < 1 dose in 10day period or 1 dose/week	54
**Williams**	2007	West Indies	96	Clinic	54.2	35.6	95	patient or provider	≥95, no. Doses taken/no. prescribed in past 7 days	87.7-patient, 87.0-provider
**Abstracts**										
**Dahab**	2006	South Africa	69	Workplace ART programme	1%	43.1	Unknown	patient	<1 log drop in viral load at 6 weeks after treatment start	86
**Shah**	2006	India	279	Private clinic	27		Unknown	patient	>95, doses missed in past 4 days	73
**Warley**	2006	Brazil	71	clinic	58	37.9	Unknown	patient	>95, doses missed in past 4 days	70.4
**Summary (median, range)**					**51.5 (1**–**77)**	**37.9 (34.6**–**47.8)**	**95 (63.2**–**100)**			**73.6 (25**–**88)**

**Table 4:** Characteristics of included longitudinal papers [n = 3, (43–45)] and conference abstracts [n = 3, (49–51)].

Author	Year	Country	N	Setting	Female (%)	Median Age, y	Response Rate	Follow-up (FU)	Assessor	Adherence, % threshold for measurement	Adherence Proportion (%)
**Bonolo**	2005	Brazil	306	Public referral hospital	35	35	73.4%	Median overall FU time: 247 days	patient	≥95%, number doses taken in past 3 days	cumulative: 36.9%
**Erah**	2008	Nigeria	102	HIV treatment centre	64	mean: 36.3 (7.9)	81.6	Unknown	patient	≥95%, number doses taken in past 30 d	58.1
**Orrell**	2003	South Africa	289	University HIV clinic	43	33.4 (8.7)	96.2	87.5% after 4 wk; 83.7% after 48 wk	pharmacy refill and pill count	≥90, medication dispensed minus pills returned/no. Pills prescribed over 48 weeks	63
**Abstracts**											
**Abah**	2006	Nigeria	130	Teaching hospital	N/A	N/A	N/A	N/A	patient and pharmacy	≥95, % of doses prescribed over 6 month period	85.1
**Darder**	2004	South Africa	192	Clinic	N/A	N/A	N/A	N/A	patient	≥95, % of doses	88
**Sidle**	2007	Kenya	7381	clinics					patient	100	77
**Summary (median, range)**					**43 (35**–**64)**	**35 (33.4**–**36.3)**	**81.6 (73**–**96.2)**				

N/A: Not Available.

Sixteen studies (13 papers, 3 abstracts) were conducted in Africa [26]–[32], [35], [39], [41], [44], [45], [46], [49]–[51]. Seven studies (6 papers, 1 abstract) were conducted in Central and South America [33], [34], [37], [38], [42], [43], [48], with the majority in Brazil [33], [34], [37], [43], [48]. Two studies (1 paper, 1 abstract) [36], [47] were conducted in India and 1 study in China [40]. Since the most recent systematic review which examined factors affecting adherence [15], eleven cross-sectional (64.7%) [26], [27], [29], [31], [35]–[37], [39], [40], [42] and 2 longitudinal studies (66.7%) have been published [44], [51].

Included studies reflect a diverse range of settings and study populations. Studies were conducted primarily in public, teaching, or referral hospitals (n = 9), public outpatient or community-based clinics (n = 10), and specialist/HIV clinics or treatment centres (n = 6), although private clinics (n = 3) and a workplace ARV programme (n = 1) were also reported. The proportion of women included in cross-sectional studies ranged from 1–77% (median 51.5%) and between 35–64% (median 43%) in longitudinal studies. The median age was 37.9 and 35 for participants in cross-sectional and longitudinal studies respectively. The response rate was unknown for 4 cross-sectional papers [28], [32], [35], [37] and all conference abstracts.

### Adherence Threshold Measurements

Twenty-two studies (84.6%) assessed adherence using patient-reported adherence levels over a specified period. One study used pharmacy claims and three used a combination of patient and clinician/provider assessment. Twelve papers [28]–[34], [40]–[42], [43], [44] and 4 abstracts [47]–[50] defined adherence as greater or equal than 95% during the measurement period, which ranged from 2 days to 6 months. Five studies [26], [35], [38], [39], [51] defined adherence as being 100% during the measurement period. Three studies [36], [37], [45] assessed adherence as greater than 90% over the measurement period. Median adherence proportions in cross-sectional studies were 74.3% (Range 25–88%) for papers alone and 73.6% when the three conference abstracts were added. While the median adherence levels among the 3 longitudinal studies were 58.1%, it increased to 70% when the three conference abstracts were added. Overall range differed little from the cross-sectional studies (36.9–88%).

### Financial, Human, and Social Capital Factors Affecting Adherence: (Tables 5–9)

**Table 5:** Financial, Human, Social Capital factors associated* with adherence or non-adherence to antiretroviral therapy in included cross-sectional papers [n = 7, (26–32)].

	Financial Capital			Human Capital			Social Capital				Other
Author/Year	Financial constraints/ARV payment	Household Income	Distance to clinic/transport costs	Education	Employment Status	Food-related restrictions	Marital Status	Household size	Social support	Fear of Stigma/non-disclosure	Reasons for missing doses
**Aboubacrine/2007**					work with no stable salary vs. no work associated with adherence: OR: 3.15 (1.15–11.13)			Having children vs. not associated with adherence OR: 2.36 (1.08–5.15)			
**Boyer/2005**	Difficulty buying ARV and reporting high adherence: OR: 0.24 (0.15–0.4)										
**Byakika-Tusiime/2005**		Monthly < $US 50 associated with non-adherence 0R: 2.42 (1.24–4.0), AOR: 2.77 (1.64–4.67)		Education level attained, ns	ns		being single associated with non-adherence: OR: 1.19 (0.73–1.95) AOR: 2.93 (1.32–6.5)		ns		lack of money (72.4%), away from home (11.2%)
**Carlucci/2008**			Travel duration: ns Transport cost: ns							Stigma vs. none, ns	
**Iliyasu/2005**				formal vs. no formal education associated with adherence: OR: 3.97 (1.75–9.24)							lack of funds (15.8%)
**Malangu/2008**				Having a tertiary education vs. other, ns	Being employed vs. other, ns	Eating well associated with adherence (p = 0.03)					Away from home (15.6%)
**Nachega/2004**		Ns		Ns	Being employed vs. unemployed: ns					Fear of stigma from partner vs. no associated with adherence OR: 0.13 (0.02–0.70)	being away (30%), stigma (75%)

*OR: Odds Ratio, AOR: Adjusted Odds Ratio, (n_1_–n_2_): 95% Confidence Intervals, sig: significant, ns: not significant.

**Table 6 pone-0018948-t006:** Financial, Human, Social Capital factors associated* with adherence or non-adherence to antiretroviral therapy in included cross-sectional papers [n = 4, (33–36)].

	Financial Capital			Human Capital			Social Capital				
Author/Year	Financial constraints/ARV payment	Household Income	Distance to clinic/Transport costs	Education	Employment Status	Food-related restrictions	Marital Status	Household size	Social support	Fear of Stigma/non-disclosure	Reasons for missing doses
**Nemes/2004**				0–2 years schooling associated with non-adherence OR: 1.51 (1.12–2.02), AOR: 1.48 (1.16–1.89)							
**Pinheiro/2002**		Monthly income, ns		≥8 years of schooling vs. 0–4 associated with adherence AOR: 2.26 (1.02–5.02)							
**Ramadhani/2007**	Paying for treatment associated with non-adherence OR: 4.9 (1.92–25.9), AOR: 23.5 (1.2–444.4) Sacrifice health for other needs OR: 20.7 (3.9–110.3), AOR: 19.8 (3.1–127.8)		Walking time to the clinic associated with non-adherence OR: 1.2 (1–1.5)							Disclosure of HIV associated with non-adherence OR: 0.23 (0.05–1.1), AOR: 0.16 (0.02–1.1)	
**Sarna/2008**	free ARV vs. paid out-of- pocket associated with non-adherence: 5.71 (2.94–11.10), AOR: 4.05 (1.42–11.54)			< 5 years education vs. university associated with non-adherence: OR: 4.28 (1.49–12.33), 6–12 years, OR: 2.83 (1.29–6.19)	Unemployed vs. employed associated with non-adherence: OR: 2.35 (1.22–4.88). AOR: ns						travel, financial difficulties

*OR: Odds Ratio, AOR: Adjusted Odds Ratio, (n_1_–n_2_): 95% Confidence Intervals, sig: significant, ns: not significant.

**Table 7 pone-0018948-t007:** Financial, Human, Social Capital factors associated* with adherence or non-adherence to antiretroviral therapy in included cross-sectional papers [n = 6, (37–42)].

	Financial Capital			Human Capital			Social Capital				
Author/Year	Financial constraints/ARV payment	Household Income	Distance to clinic/Transport costs	Education	Employment Status	Food-related restrictions	Marital Status	Household size	Social support	Fear of Stigma/non-disclosure	Reasons for missing doses
**Silva/2009**		Higher income associated with adherence: p = 0.08, AOR: 2.33 (1.17–4.66)		8 years of schooling vs. 11 years, ns							
**Stout/2004**			Difficulty finding transport vs. other associated with non-adherence OR: 6.3, (1.5–26.9)			Difficulty taking meds on empty stomach vs. other OR: 6.7 (1.3-35.7)					travel away from home: 17%
**Uzochukwu/2009**		Ns	Living 20+Km associate with adherence, p = 0.038	formal education associated with non-adherence (p = 0.0394)			being single associated with non-adherence p = 0.02				Cost and transport (30.1%), sold drugs because need money (28.2%)
**Wang/2007**				HIV knowledge associated with adherence OR: 5.59, (2.48–12.57), AOR: 3.20, (1.24–8.26)					Support as reminder tool associated with adherence: OR: 4.22, (1.90–9.39), AOR: 3.49, (1.36–8.96)		Community/social activities (16.3), food restrictions (11.2%), stigma (14.3%)
**Weiser/2003**	Cost as a barrier to treatment associated with adherence: OR: 0.15 (0.06–0.35), AOR: 0.11 (0.04–0.30)			Incomplete secondary ed compared to complete associated with adherence OR: 3.87 (1.21–12.40)			ns			Disclosure to others associated with adherence OR: 3.55 (0.91–13.92)	Financial difficulties (48%), travelling (12%), distance to clinic (5%), stigma (3%)
**Williams/2007**				positive, ns	positive, ns						No food, social problems

*OR: Odds Ratio, AOR: Adjusted Odds Ratio, (n_1_–n_2_): 95% Confidence Intervals, sig: significant, ns: not significant.

**Table 8 pone-0018948-t008:** Financial, Human, Social Capital factors associated* with adherence or non-adherence to antiretroviral therapy in included cross-sectional abstracts [n = 3, (46–48)] and summary of associations in all cross-sectional studies [n = 20, (26–42, 46–48)].

	Financial Capital			Human Capital			Social Capital			
Author/Year	Financial constraints/ARV payment	Household Income	Distance to clinic/Transport costs	Education	Employment Status	Food-related restrictions	Marital Status	Household Size	Social Support	Fear of stigma/non-disclosure
**Dahab/2006**				Educated vs. other associated with adherence OR: 2.4 (1.2–4.7)						
**Shah/2006**				Positive association, sig.						Fear of stigma, Negative association, sig.
**Warley/2006**				ns	ns				ns	
**SUMMARY of associations with adherence across all cross-sectional studies (n = 20)**	n = 4 Positive: 1 Negative: 3	n = 5 Positive: 2 No assoc: 3	n = 4 Positive:1 Negative:2 No assoc: 1	n = 15 Positive: 7 Negative: 2 No Assoc: 6	n = 7 Positive: 2 No Assoc: 5	n = 2 Negative:2	n = 3 Negative (single): 2 No assoc: 1	n = 1 Positive: 1	n = 3 positive: 1 No assoc: 2	n = 5 No Assoc: 1 Positive (disclosure):3 Negative (stigma):1

*OR: Odds Ratio, AOR: Adjusted Odds Ratio, (n_1_–n_2_): 95% Confidence Intervals, sig: significant, ns: not significant.

**Table 9:** Financial, Human, and Social capital associated* with adherence or non-adherence to antiretroviral therapy in included longitudinal studies and summary of all associations [n = 6, (43–45, 49–51)].

	Financial Capital			Human Capital			Social Capital			Other
**Author/Year**	Financial constraints/payment of ARV	Household Income	Distance from Clinic	Education	Employment Status	Food-Related Restrictions	Marital status	Household size	Social support	Reasons for missing doses:
**Bonolo/2005**		Individual ≤US$80 vs. greater, associated with non-adherence RH: 1.61 (1.08–2.39)		≤4 years school vs. > 8 associated with non-adherence RH: 1.80 (1.08–2.29)	Unemployed vs. employed associated with non-adherence: RH: 2.16 (1.20–3.91), ARH: 2.17 (1.19–3.96)				Does not participate in religious activities vs. regular activity associated with non-adherence ARH: 2.27 (1.58–3.25)	
**Erah/2008**				None or primary education associated with non-adherence OR: 1.81 (1.25–2.51), AOR: 2.23 (1.02–2.89)				ns		poor financial status and inadequate family support (15.9%), occupational factors (25%)
**Orrell/2003**	ns	ns				Restrictions associated with adherence: ns				
**Abstracts**										
**Abah/2006**			ns	ns	ns		Married associated with adherence, p = 0.02			lack of money (17.1%)
**Darder/2006**				Positively associated with adherence, sig.						
**Sidle/2007**				Level of education associated non- adherence: AOR: 0.96, p = 0.0269				More children associated adherence, AOR = 1.19, p = 0.0352		
**Summary of associations with adherence**	n = 1, No assoc:1	n = 2 Positive: 1 No assoc: 1	n = 1, No assoc: 1	n = 5 Positive: 4 No Assoc. : 1	n = 2 Positive: 1 No assoc.: 1	n = 1 No assoc.: 1	n = 1 Positive (married): 1	n = 2 Positive: 1 No Assoc.: 1	n = 1 Positive: 1	

*OR: Odds Ratio, AOR: Adjusted Odds Ratio, RH: Relative Hazard, ARH: Adjusted Relative Hazard.

#### Financial Capital

Five studies, 4 cross-sectional [27], [35], [36], [41] and 1 longitudinal [45], measured the association between financial constraints/ability to pay for treatment and adherence. Two reported lower levels of adherence associated with increasing financial difficulties [27], [41]. One study reported that the need to sacrifice health to pay for other resources such as housing was associated with non-adherence (Odds Ratio (OR): 19.8, 95% Confidence Intervals (CI): 3.1–122.7) [35]. One study reported that non-adherence was associated with having access to free treatment (OR: 4.05, 95% CI: 1.42–11.54) [36] while another reported that the association between financial constraints and adherence was not-significant when examined over time [45].

Five cross-sectional [28], [32], [34], [37], [39] and 2 longitudinal [43], [45] studies examined the association between household income and adherence, 4 of which demonstrated a non-significant association [32], [34], [39], [45]. One study reported an increase in non-adherence associated with a monthly income of <$50 US (Adjusted OR (AOR): 2.77, 95% CI: 1.64–4.67) [28] while one cross-sectional and one longitudinal study reported that adherence was associated with an increase in household (AOR: 2.33, 95% CI: 1.17–4.66) [37] or individual income (Relative Hazard (RH): 1.61, 95% CI: 1.08–2.39) [43].

Five studies (4 cross-sectional [29], [35], [38], [39], 1 longitudinal [49]) examined how distance from the clinic and the ability to pay for transport impacted adherence. One large study showed a non-significant association [29]. One study demonstrated that living more than 20 km away was positively associated with better adherence [39], while two studies demonstrated a negative association, with non-adherence increasing with distance (OR: 1.2, 95% CI 1.0–1.5) [35] or difficulty finding transport (AOR: 6.3, 95% CI: 1.5–26.9) [38].

There was a statistically significant positive association demonstrated between overall financial livelihood and adherence proportions (exponentiated beta coefficient =  1.53, 95% CI: 1.03–2.29, p = 0.04).

#### Human Capital

Fifteen cross-sectional [28], [30]–[34], [36], [37], [39]–[42], [46]–[48], and five longitudinal [43], [44], [49]–[51] studies examined the association between education and adherence. One study examined HIV knowledge and reported that increasing education and knowledge about HIV was associated with adherence (AOR: 3.20, 95% CI: 1.24–8.26) [40]. Six cross-sectional [30], [33], [34], [36], [46], [47] and four longitudinal studies [43], [44], [50], [51] reported a positive association between education and adherence. Two cross-sectional studies reported a negative association: one reporting that a formal education was associated with non-adherence [39] and another reporting higher levels of adherence among individuals who had not completed secondary school compared to those who had (OR: 3.87, 1.21–12.40) [41].

Nine studies, seven cross-sectional [26], [28], [31], [32], [36], [42], [48] and two longitudinal [43], [49], examined the association with employment status. Of these, one cross-sectional [26] and one longitudinal [43] study reported a positive and significant association between employment and adherence.

Three studies, 2 cross-sectional [31], [38] and one longitudinal [45], measured the association with food-related restrictions. One study reported that adherence was positively associated with eating well [31] while another demonstrated that non-adherence was associated with not having enough food to take with medications (OR: 6.7, 95% CI: 1.3–35.7) [38].

No statistically significant association between human capital and adherence was found (exponentiated beta coefficient = 1.04, 95% CI: 0.71–1.53, p = 0.81).

#### Social Capital

Four studies (3 cross-sectional [28], [39], [41], 1 longitudinal [49]) examined the role of marital status: one study demonstrated that being single was positively associated with adherence (AOR: 2.93, 95% CI: 1.32–6.5) [28] while another demonstrated that being single was negatively associated with adherence [39]. Being married was positively associated with adherence in one longitudinal study [49].

One cross-sectional (OR: 2.36, 95% CI: 1.08–5.15) [26] and 1 longitudinal study (AOR: 1.19, p = 0.0352) [51] reported a positive association between household size, specifically the number of children, and adherence.

Three studies, 3 cross-sectional [28], [40], [48] and one longitudinal [43]), examined the role of social support: One study found that using support networks as reminder tools was positively associated with adherence (AOR: 3.49, 95% CI: 1.36–8.96) [40]. A longitudinal study reported that not participating in any religious activities was associated with non-adherence (Adjusted RH (ARH): 2.27, 95% CI: 1.58–3.25) [43].

Fear of stigma and disclosure of HIV status was examined in 5 cross-sectional studies [29], [32], [35], [41], [47]. Two studies reported that stigma was negatively associated with adherence to ARVs [32], [47].

Overall social livelihood and adherence were positively associated but not significant for this set of studies (exponentiated beta coefficient: 1.79, 95% CI: 0.63–5.08, p = 0.21).

### Patient-report reasons for missing doses/non-adherence

Ten cross-sectional studies [28], [30]–[32], [36], [38]–[42] and two longitudinal studies [44], [50] reported additional patient-identified barriers to treatment adherence (i.e., reasons for missing doses). Reported barriers included: financial difficulties (n = 7) [28], [30], [36], [39], [41], [44], [50], being or travelling away from home (n = 7) [28], [31], [32], [36], [38], [39], [41], fear of stigma (n = 3) [32], [40], [41], the need to participate in social activities (n = 2) [40], [42], food restrictions (n = 2) [40], [42], inadequate family support (n = 1) [44] and occupational factors (n = 1) [44].

## Discussion

The diversity of studies included in this review and the lack of consistency between them suggests that the literature on livelihood and HIV treatment outcomes is still in its infancy. Studies were conducted in numerous settings and the measurement tools used to assess and define both livelihood factors and adherence varied substantially. Adherence proportions ranged from 25% to 88% and were tested for associations with ten different livelihood factors related to financial, human, or social capital.

Education level was the most commonly measured livelihood factor. While almost all studies indicated that a higher level of education was associated with adherence, two studies reported a negative association [39], [41]. Higher levels of education have previously been associated with increased risky behavior and risks of HIV infection [52]. The reasons for this remain unclear. Talam et al. (2008) argue that better educated patients may be too busy with their professional activities to take their pills regularly [53]. In contrast, others have argued that greater access to information as a result of higher education, likely helps individuals to make more informed decisions about the need to remain adherent [44], [54]. Higher educated patients may also be better equipped to plan, organize, and integrate new realities into their daily lives [55]. Furthermore, education level has also been considered an important determinant of self-efficacy which previously has been positively associated with adherence to ARVs [34], [55].

Financial capital was one key factor impacting on adherence to ARVs and the only type of capital which demonstrated a significant association with adherence. The inability to afford medication was one of the most frequently reported reasons for non-adherence both in included studies [27], [28], [35], [39], [41] as well as in others [56]–[58]. While access to free ARVs was associated with non-adherence in one study [36], individuals receiving free treatment may be more likely to be highly impoverished and facing numerous obstacles (e.g., lack of food, shelter) which impact on their ability to adhere [59]. Importantly, user fees and charges for treatment are widespread in resource-poor settings [39] although it has been suggested that optimal levels of adherence can be achieved with access to subsidized ARVs [41], [45]. A 2005 meta-analysis focused on ARV programmes in resource-poor settings reported that, in fact, when medications were provided free-of-charge, there was a higher probability of achieving adherence and undetectable viral loads compared to situations where patients were required to pay for treatment [18].

Even in the context of free drugs however, the cost of transportation to obtain ARVs can still remain a barrier to adherence. Research from sub-Saharan Africa has demonstrated that patients often have to choose between using their limited income on paying for transportation to the clinic versus being able to adequately feed their families [60]. As a result, individuals may miss their scheduled clinic appointments and thus not receive their ARVs at the regular time intervals critical for optimal adherence [38], [61]. Therefore, increasing access to affordable transportation as well as expanding the number and location of ARV clinics may help to facilitate HIV treatment adherence [38], [62].

Findings related to food-related restrictions i.e. inability to take medications on an empty stomach, and greater adherence when eating well, were also associated with adherence in two studies. Food insecurity has been well-documented in Africa and has been linked with decreased adherence to ARVs and poor clinical outcomes [61], [63]–[65]. Medication-related food restrictions place an additional burden on patients, in many ways, increasing the complexity of the treatment regimen itself [62], [66]. The perception that ARVs need to be taken with food may lead to non-adherence [40], [42], [67], [68] suggesting that access to adequate food via self-production or, at the very least, food supplements, bolsters human capital, recognizing that taking pills on an empty stomach may lead to gastrointestinal upset.

Social stability and social capital have both been associated with medication adherence in various settings [69]–[73]. Importantly, social support can take the form of direct reminders, financial help, and emotional backing [71]. Qualitative research from South Africa suggests that treatment supporters (i.e., clinic buddies) are a valuable aid in promoting adherence [74]. As identified in this review, social factors such marital status and having children can impact on adherence. The desire to be alive and be able to support their families and see their children grow up may be a strong motivator for patients to adhere [39], [69], [71], [75]. However, disclosure to one's sexual partner has been recognized as a double edged sword [74]- it has the potential to yield much needed social support [35] but may also result in stigmatization, discrimination, and potentially abandonment [74], [76], [77]. This may partly explain why adherence was higher for single individuals in one study [28]. Issues of stigma and discrimination related to HIV/AIDS remain a real concern in many settings [78] and may lead to social isolation, limit sources of social capital, and undermine relationships that are essential for survival [70], [78]. Further research in this area is still needed to help elucidate the type and nature of social capital that impacts on adherence across settings.

### Limitations

Limitations in our review reflect the quality and nature of included studies. While our search was extensive and we did seek clarification from various study authors, it is possible we missed unpublished studies measuring the association between relevant livelihood factors and adherence. There is no gold standard for measuring adherence -patient recall and pill count, both commonly used, have inherent biases in their use [79]–[81]. For example, there is a tendency for self-reported adherence to be positively skewed (i.e., patients overestimating adherence levels) increasing the risk of patient misclassification [79]. While reporting bias, specifically social desirability bias, may have a profound impact here, other influences may include question misinterpretation and issues of recall [82]. Additionally, the lack of methodological standards makes assessments and comparisons between levels of adherence difficult. As such there remains the need for validation of adherence monitoring tools capable of measuring real-time behaviour of patients in various settings [81]. While numerous livelihood factors were examined, few were measured using standardized or validated instruments for particular constructs, again reflecting the dearth of research conducted in this area to-date and perhaps the lack of experience among clinical investigators with use of standard measures more out of social science traditions.

Unmeasured or unidentified features of included studies may also have a large impact on either apparent or real adherence [15]. Detailed population descriptions (e.g., education level) and the regional and political conditions under which a study was conducted would assist interpretation of future studies in this field. For example, patients with access to private or non-governmental health services may have additional benefits including better access to laboratory equipment and testing [43] which may ultimately affect treatment outcomes. Furthermore, patients in care and on ARVs may differ meaningfully from patients who either lack access to ARVs or who refused treatment. The experiences of livelihood insecurity for these individuals may, in part, explain why they are not or did not remain in care, but such populations were rarely included. Finally, the majority of included studies were cross-sectional in study design, limiting the ability to establish temporal causality (i.e., livelihood to adherence) validly.

### Conclusions

We found only one significant association that was consistent across settings. We demonstrated a positive association between financial capital and adherence whereas no statistically significant relationship was found for human or social capital and adherence. Importantly, the included studies reflect a range of experiences in the association between various livelihood factors and adherence to ARVs. This heterogeneity and diversity can also be considered an important strength of this review. More longitudinal studies that can effectively measure and monitor the dynamic interactions between livelihood security and adherence across settings are needed. Linked to these could be additional qualitative work able to explore the lives and treatment challenges of PLWHA [83]. Both study designs are essential for understanding adherence, the way adherence changes over time, and the reasons for non-adherence. Through their incorporation in structural policies or programs, findings from such research can contribute to improved patient outcomes [84]. Furthermore, clinicians and other health providers can actively work with their patients and help them to prioritize adherence while addressing potential obstacles to care [84], [85]. As many of these obstacles and challenges lie beyond the control of the individual patient, addressing adherence in low- and middle-income settings, therefore, may require eliminating or lowering user fees and patient costs, bringing care closer to the patients, and implementing community-based livelihood development strategies.
